# A Graphene Oxide-Based Fluorescent Aptasensor for the Turn-on Detection of CCRF-CEM

**DOI:** 10.1186/s11671-017-2403-3

**Published:** 2018-04-01

**Authors:** Jie Tan, Zongqiang Lai, Liping Zhong, Zhenghua Zhang, Rong Zheng, Jing Su, Yong Huang, Panpan Huang, Hui Song, Nuo Yang, Sufang Zhou, Yongxiang Zhao

**Affiliations:** National Center for International Research of Biological Targeting Diagnosis and Therapy, Guangxi Key Laboratory of Biological Targeting Diagnosis and Therapy Research, Collaborative Innovation Center for Targeting Tumor Diagnosis and Therapy, Guangxi Medical University, Nanning, Guangxi 530021 China

**Keywords:** Aptamer, Graphene oxide, Leukemia, CCRF-CEM

## Abstract

A convenient, low-cost, and highly sensitive fluorescent aptasensor for detection of leukemia has been developed based on graphene oxide-aptamer complex (GO-apt). Graphene oxide (GO) can absorb carboxyfluorescein-labeled Sgc8 aptamer (FAM-apt) by *π*-*π* stacking and quench the fluorescence through fluorescence resonance energy transfer (FRET). In the absence of Sgc8 target cell CCRF-CEM, the fluorescence is almost all quenched. Conversely, when the CCRF-CEM cells are added, the quenched fluorescence can be recovered rapidly and significantly. Therefore, based on the change of fluorescence signals, we can detect the number of CCRF-CEM cells in a wide range from 1 × 10^2^ to 1 × 10^7^ cells/mL with a limit of detection (LOD) of 10 cells/mL. Therefore, this strategy of graphene oxide-based fluorescent aptasensor may be promising for the detection of cancer.

## Background

Leukemia is an aggressive and common malignant hematologic disease, which is a threat to the survival of human beings and health, especially for children and adolescents [1, 2]. It affects not only the body’s normal hematopoietic cells but also the bone marrow, as well as the immune system [3–5]. Therefore, the early diagnosis of leukemia for the treatment and the improvement of the quality of life of patients is essential. At present, the commonly used method for detecting leukemia is taking peripheral blood cells and bone marrow, after that many kinds of analysis [6], including cell morphology, cytochemistry [7–9], immunophenotype [10, 11], immunohistochemical [12, 13], and aptamer-based flow cytometry [14, 15], have been carried out. These methods can detect leukemia cells, but they still have many shortcomings such as high cost, low sensitivity, and being complicated. Therefore, it is very urgent to find a low-cost, highly sensitive, and simple method for detecting leukemia.

Aptamers, which are short single-stranded DNA (ssDNA) or RNA, were screened by in vitro screening of systematic evolution of ligands by exponential enrichment (SELEX) [16, 17]. Based on the special tertiary structures, aptamers have robust binding affinity and high specificity with targets, including small organic molecules, proteins, and even cells [18–20]. Moreover, aptamers also have the characteristics of being easily synthesized and modified so that they are widely used as cancer detection probes [21]. Functionalized nanomaterials based on aptamers for detection of cancer are also hotspots in recent years [22, 23], such as quantum dots and silica nanoparticles [24].

Graphene oxide (GO), as a novel two-dimensional planar carbon nanomaterials, has received substantial attention owing to its unique properties including good aqueous solubility [25], large specific surface area, and excellent fluorescence quenching ability [26, 27]. Based on these properties, GO is considered to be an excellent energy receptor in fluorescence resonance energy transfer (FRET), which makes GO have a broad application prospect in fluorescence aptasensor [28]. Moreover, GO can bind to aptamers by *π*-*π* stacking interactions, but not with double-stranded DNA or aptamer-target complexes [19, 29, 30]. Hence, the graphene-based aptamer sensor can improve the stability of the aptamer compared to the free aptamer probe [31].

At present, a great deal of researches reported that the strategy of graphene oxide-based fluorescent aptasensor for detection target is feasible [21, 32]. Nevertheless, few studies have been carried out using a GO-based aptasensor for leukemia cells, so far. Here, we designed a new strategy for the signal ‘turn-on’ detection of leukemia cells based on GO and carboxyfluorescein-labeled Sgc8 aptamer (FAM-apt). GO and aptamer were used as a fluorescence quencher and target agent, respectively. In the absence of leukemia cells, GO can interact with FAM-apt and quenched almost all the fluorescence, and the detection signal turned off. However, when the target cells are present, the aptamers actively target cells and fall off from GO, resulting in fluorescence recovery in the detection system, and the detection signal turned on. Therefore, the target cell concentration can be measured correspondingly according to the change in fluorescence intensity.

## Methods

### Reagents

The FFAM-apt with a sequence of 5′-FAM-AT CTAACTGCTGCGCCGCCGGGAAAATACTGTACGGTTAGA-3′ was synthesized by the Sangon Biotech Co., Ltd. (Shanghai, China). In this work, self-regulating Tris-HCl buffer was employed, including 20 mM Tris-HCl (pH 7.4), 5 mM MgCl_2_, and 100 mM NaCl. The aptamers used in this experiment were dissolved by Tris-HCl buffer. Graphene oxide powder was purchased from the Xianfeng Nano Materials Tech Co., Ltd. (Nanjing, China). All solutions were prepared with ultrapure water of 18 MΩ purified from a Milli-Q purification system (Millipore, Bedford, MA, USA).

### Cells

CCRF-CEM (human acute leukemic lymphoblast cell lines), Ramos (human Burkitt’s lymphoma cell lines), 293T (human embryonic kidney cell lines), and H22 (murine hepatocellular carcinoma cell lines) cell lines were purchased from the Cell Bank of the Chinese Academy of Sciences (Shanghai, China). All cell lines were cultured at 5% carbon dioxide and 37 °C, and the medium of 1640 contains 10% fetal bovine serum (FBS; HyClone) and 100 U/mL penicillin-streptomycin (Gibco, Grand Island, NY, USA).

### Apparatus

All fluorescence spectra and fluorescence intensity were measured and recorded by an F-7000 fluorescence spectrophotometer (Hitachi Company, Tokyo, Japan). A 700-μL quartz cuvette was used to hold the sample solution. Owing to the characteristic peak wavelengths of carboxylfluorescein (FAM), the luminescence intensity was monitored by exciting the sample at 490 nm and measuring the emission at 518 nm.

All the atomic force microscopy (AFM) imaging was taken by a SPI3800N microscope (Seiko Instruments Industry Co., Tokyo, Japan).

Zeta potential of the GO, FAM-apt, and graphene oxide-aptamer complex (GO-apt) was determined by a nanoparticle size, zeta potential, and absolute molecular weight analyzer (Zetasizer Nano ZS, Malvern, UK).

UV-visible absorbance spectra of GO, FAM-apt, and GO-apt were recorded on NanoDrop 2000 (Thermo, USA).

### Preparation of GO-apt Fluorescent Aptasensor

The graphene oxide powder was dissolved and scattered in Milli-Q purified water and then dispersed by ultrasonic to obtain a homogeneous black solution with the concentration of 1 mg/mL. Diluting the stock solution by 20 mM Tris-HCl buffer, we obtained the concentration of 20 nM FAM-apt. And after that, 1 μL FAM-apt (10 μM) and 10 μL GO solution (1 mg/mL) as prepared were mixed and then diluted with Tris-HCl buffer to 500 μL.

### Cell Imaging

CCRF-CEM and Ramos cells were cultured for 12 h in six-well plates (5 × 10^5^ cells per well). Cells were washed two times with cold phosphate-buffered saline (PBS) and incubated with GO-apt solution at 4 °C in the dark for 30 min. Then, cells were washed three times and fixed for 20 min with 4% polyoxymethylene. Cells were washed again with PBS and stained with 4′,6-diamidino-2-phenylindole dihydrochloride (DAPI; Life Co., USA) for 5 min in the dark. Finally, cells were washed three times with PBS and examined by fluorescence microscopy (Nikon DS-Ri1; Japan).

### Detections of CCRF-CEM Cells

CCRF-CEM cells were collected by centrifugation and suspended in 1 mL of PBS. The different concentrations of CCRF-CEM cells (0 to 1.0 × 10^7^/mL) were incubated with a GO-apt fluorescent aptasensor at 4 °C in the dark for 30 min. After incubation, the CCRF-CEM cells were detected by fluorescence spectroscopy in the wavelength range of 560–500 nm. The limit of detection (LOD) is estimated based on the 3*σ* / *S* calculation, where *σ* is the standard deviation for the GO-apt solution (*n* = 10) and *S* is the slope of the linear equation [33].

### Specificity Assay

To investigate the specificity of GO-based fluorescent aptasensor, we tested the system with several different cells, including Ramos cells, H22 cells, and 293T cells. Each of the 100-μL reaction systems included 1 × 10^6^ cells.

### Statistical Analyses

Each experiment was repeated three times. The data was processed by the software SigmaPlot 12.5, and statistical analyses were performed using GraphPad Prism 6.02 (GraphPad Software, San Diego, CA, USA). The threshold of significance in all analyses was *P* < 0.0001.

## Results and Discussion

### Principle of GO-apt Fluorescent Aptasensor for Detection of CCRF-CEM

**Fig. 1:**
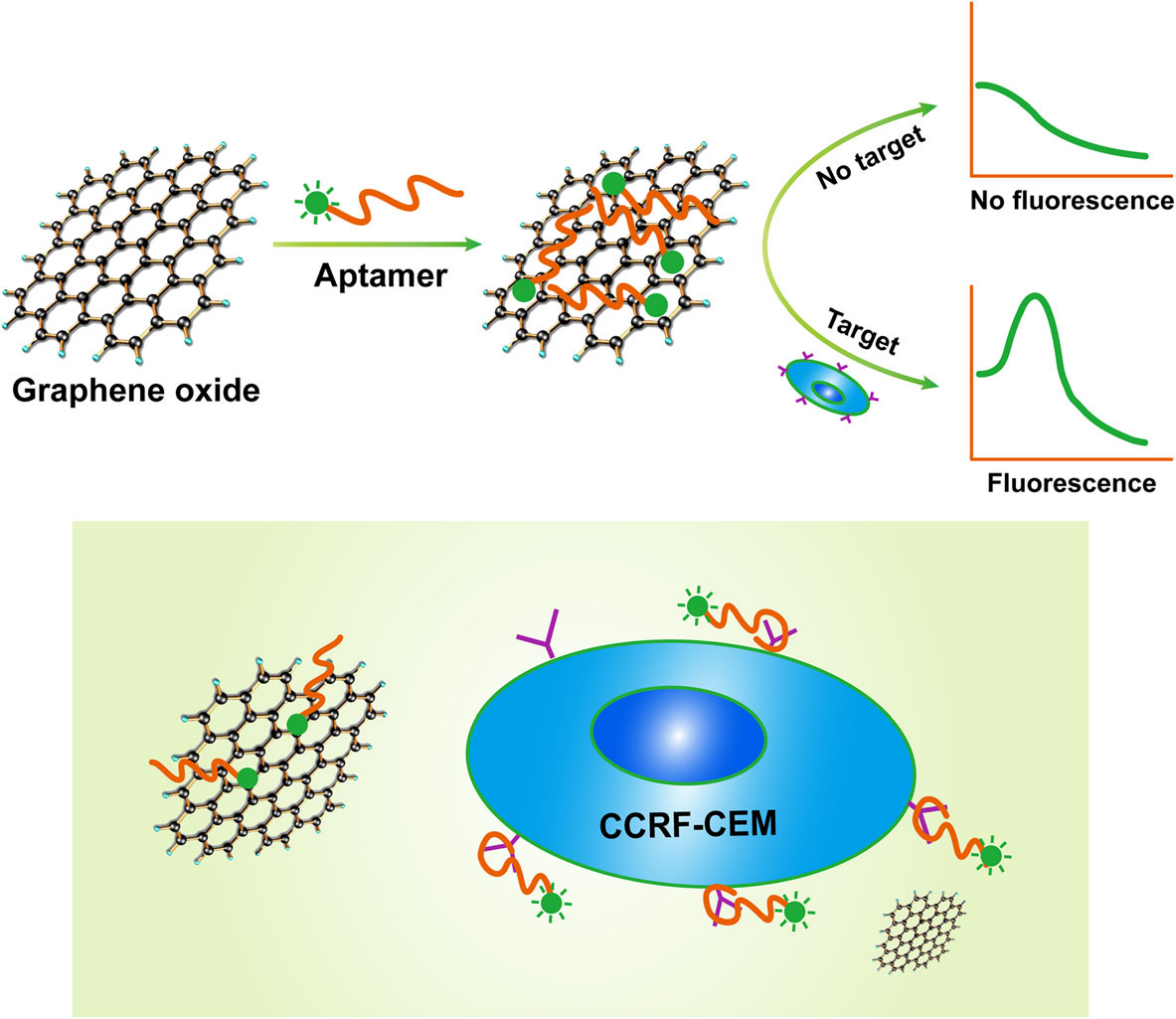
Schematic illustration of GO-apt fluorescent aptasensor for detection of CCRF-CEM cells

In this study, GO and FAM-apt were used to design a fluorescent aptasensor to detect CCRF-CEM cells. The principle of the fluorescent sensor for detection of CCRF-CEM cells is shown in Fig. 1. In the absence of CCRF-CEM cells, the FAM-modified aptamers are adsorbed onto the GO surface by *π*-*π* stacking. Since GO and the fluorophore are too close to the energy transfer, so, as a quencher, GO quenches the fluorescence of FAM. In the presence of CCRF-CEM cells, the weak binding force of the GO-aptamer allows the aptamer to fall off the GO surface and bind to the cells, causing the fluorescence restoration. Therefore, the number of CCRF-CEM cells can be detected correspondingly according to the recovery of FAM fluorescence intensity.

### Fluorescence Quenching and Recovery

**Fig. 2:**
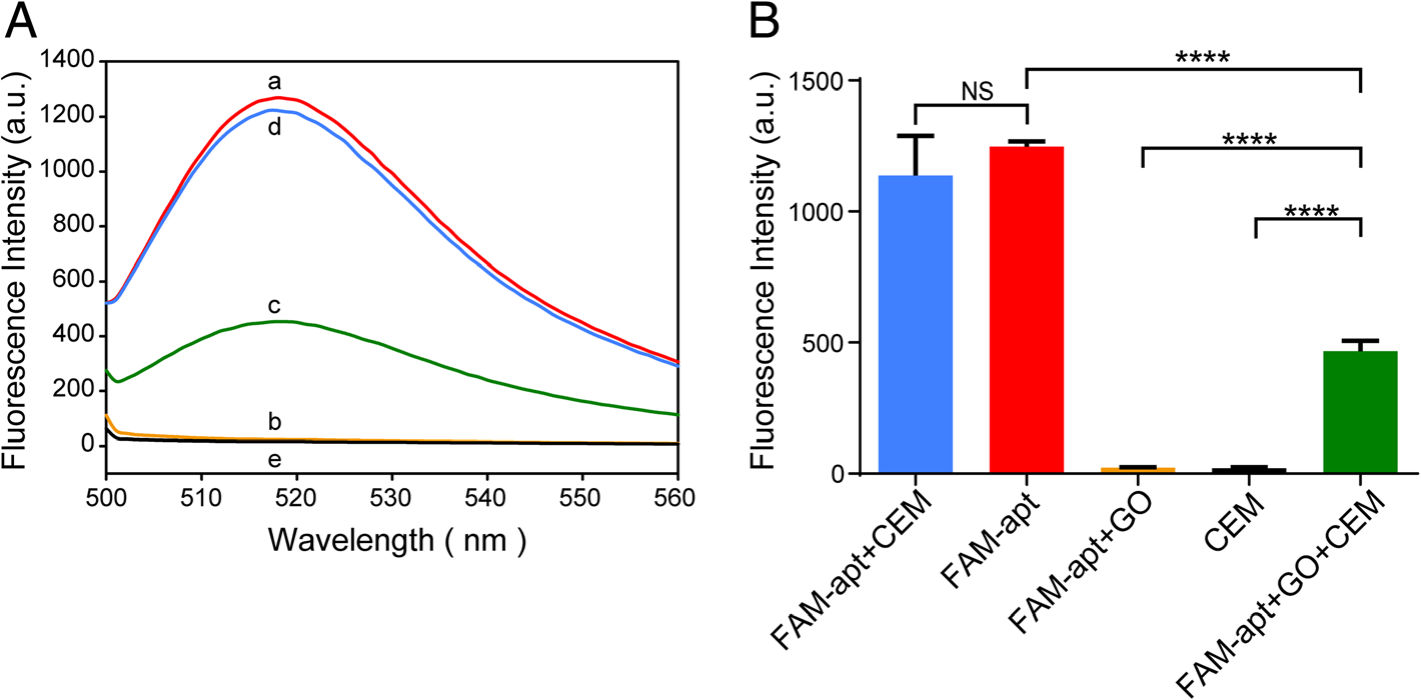
Feasibility of Go-apt detection of CEM Cells. **a** Fluorescence emission spectra of FAM-apt and CCRF-CEM cells at different conditions: (a) FAM-apt, (b) FAM-apt + GO, (c) FAM-apt + GO + CCFF-CEM, (d) FAM-apt + CCFF-CEM, and (e) CCRF-CEM; FAM-apt (20 nM); GO (25 μg/mL); CCRF-CEM (1 × 10^6^ cells). Excitation 490 nm. **b** Statistical analysis of fluorescence emission spectra of FAM-apt and CCRF-CEM at different conditions. NS not significant. ****P < 0.0001

This continuous process of quenching fluorescence of GO and returning fluorescence in the presence of CCRF-CEM cells can be observed by a fluorescence spectrophotometer. The whole process of sensing based on GO-fluorescence aptamers is shown in Fig. 2a. The fluorescence spectrum of FAM-apt in 25 nM Tris-HCl buffer presents strong fluorescence intensity thanks to the presence of the FAM (Fig. 2a, curve a). However, upon the addition of GO, the fluorescence intensity was remarkably reduced (Fig. 2a, curve b), indicating that GO was able to efficiently quench fluorescence when GO and the aptamers were close to each other and adsorbed together. Surprisingly, when 5 × 10^6^ CCRF-CEM cells were added, the quenched fluorescence was able to recover in time (Fig. 2a, curve c). Nevertheless, the fluorescence intensity of FAM-apt without GO conjugation has no obvious change when CCRF-CEM cells were added (Fig. 2a, curve d). CCRF-CEM is a non-fluorescent cell (Fig. 2a, curve e); therefore, fluorescence recovery is mainly due to the dissociation of the aptamer from the surface of the graphene and exposing the fluorescent group. These experiments of fluorescence quenching and recovery clearly illustrated that CCRF-CEM-aptamer complex (CEM-apt) can keep FAM-apt from being quenched by GO, and CEM has stronger binding affinity to its aptamer than GO. Thanks to the structure difference between single-stranded aptamer and CEM-aptamer complex, aptamers on the GO surface can interact with CEM and then transform to the CEM-aptamer complex. This phenomenon also clearly indicates that the binding of the CEM-aptamer complex to the aptamer is weaker than that of GO, thus allowing the aptamer to fall off the surface of GO. Since the FAM-apt is located away from the GO surface and the energy transfer efficiency is reduced, the fluorescence is restored. Statistical analysis of fluorescence emission spectra of FAM-labeled Sgc8 aptamer and CCRF-CEM was performed at different conditions (Fig. 2b).

### Characterizations of GO-apt Fluorescent Aptasensor

**Fig. 3:**
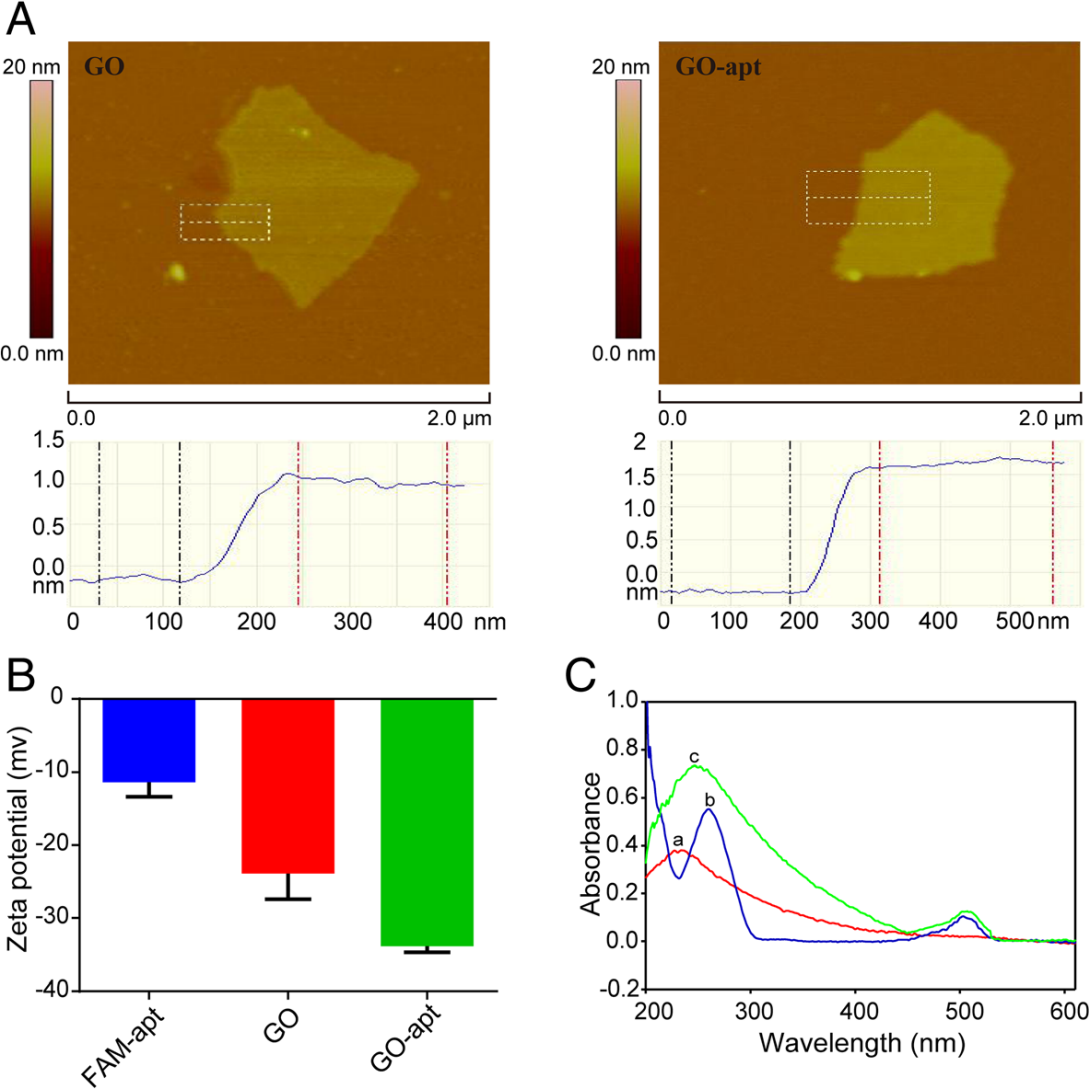
Characterization of GO-apt. **a** AFM images of GO and CEM-apt. **b** Surface zeta potential of FAM-apt, GO, and CEM-apt. Error bars indicate ± SD (*n* = 3). **c** UV-visible absorbance spectra of (a) GO, (b) FAM-apt, and (c) GO-apt

To verify the design, uniform and decentralized GO was obtained. From Fig. 3a, we know that a GO sheet with the thickness of 1.17 nm possesses a typical two-dimensional appearance by AFM. However, GO-apt with the thickness of 1.94 nm showed that FAM-apt has been absorbed to the GO surface successfully. The zeta potential of FAM-apt and GO was − 11.35 and − 23.90 mV, respectively, but when GO non-convalently interact with FAM-apt, the absolute value of zeta potential increased (Fig. 3b). These results indicated that aptasensors have been successfully constructed. From Fig. 3c, we know that GO displayed a strong absorption at 234 nm which is attributed to the *π*-π* transitions of aromatic C=C bonds. FAM-apt is characterized by absorption bands of the DNA sequence (260 nm) and FAM (503 nm), whereas the addition of GO into the solution of FAM-apt causes a red shift and the absorbance of FAM at 503 nm is increased. The possible reason is that FAM-apt is adsorbed on the GO surface, indicating electronic interactions between the two *π* systems of GO and the dyes in the ground state. Therefore, the results indicated that GO-apt has been successfully constructed.

### Fluorescence Microscopy of Cells

**Fig. 4:**
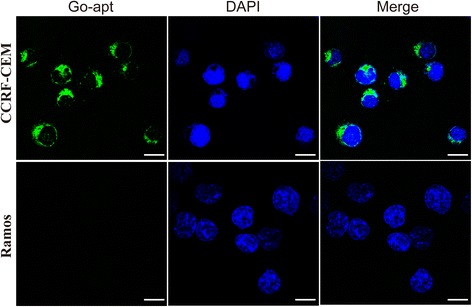
Fluorescence micrographs of CCRF-CEM and Ramos cells after mixing with GO-apt. Nuclei were stained with DAPI. Scale bars indicate 25 μm

To visualize directly the specificity of fallen FAM-apt binding at the cellular level, we incubated CCRF-CEM and Ramos cells with Go-apt and then analyzed them using fluorescence microscopy. Consistent with the fluorescence spectral experiments, FAM-apt can fall from Go-apt and then bind to CCRF-CEM cells for fluorescent staining, but not to Ramos cells (Fig. 4).

### Optimization of Experimental Conditions for Detection of CCRF-CEM

**Fig. 5:**
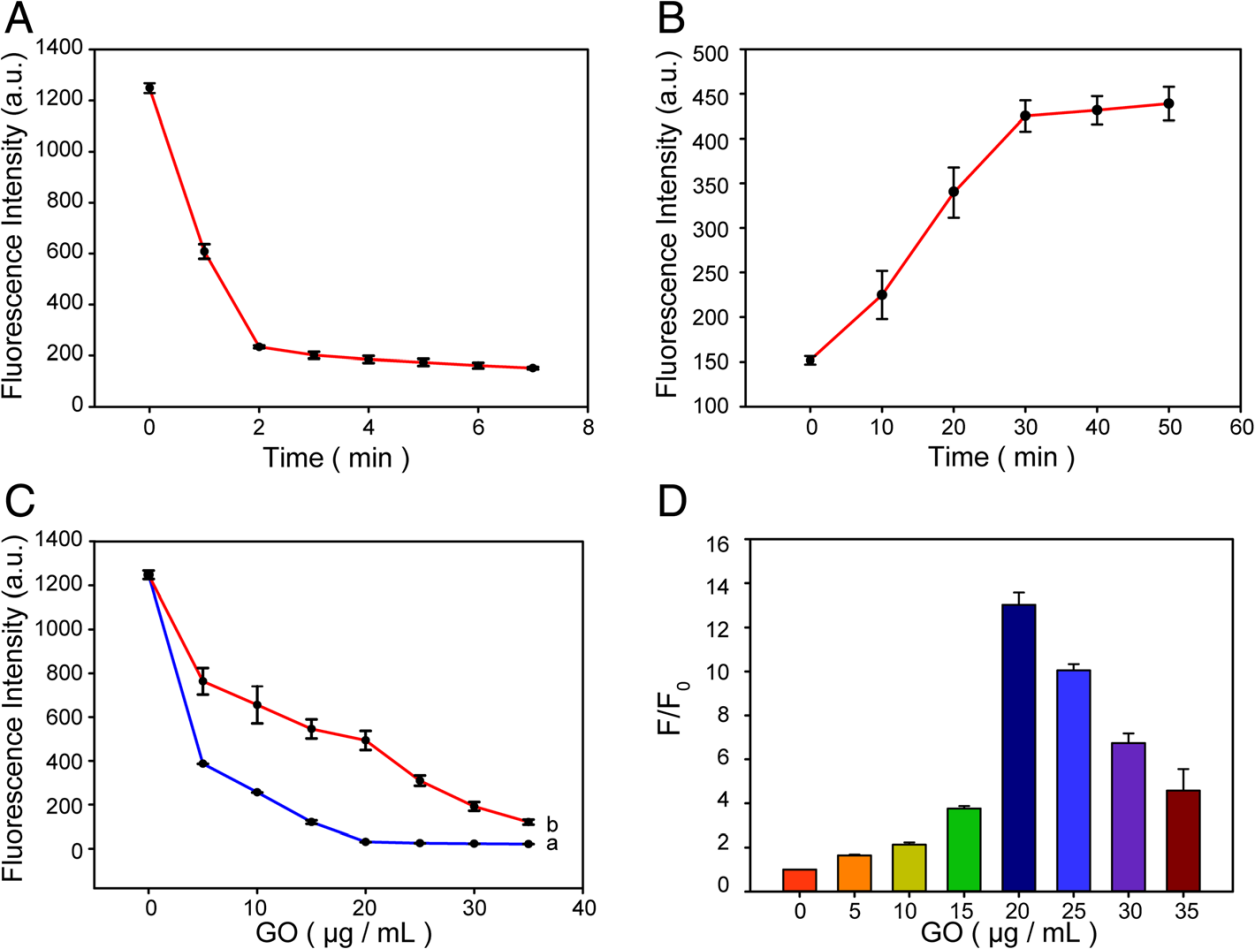
Optimization of experimental conditions. **a** Fluorescence quenching of FAM-apt (20 nM) in Tris-HCl buffer by GO as a function of time. **b** Fluorescence restoration of FAM-apt in GO solution by CCRF-CEM (1 × 10^6^) as a function of time. **c** Effect of GO concentration on the fluorescence intensity of FAM-apt in the absence (curve a) and in the presence (curve b) of 1 × 10^6^ CCRF-CEM cells. **d** The fluorescence intensity rate (*F*/*F*_0_) of FAM-apt by 1 × 10^6^ CCRF-CEM cells as a function of GO concentration. Excitation 490 nm

In order to obtain the excellent performance of the fluorescent aptasensor, the time of fluorescence quenching and recovery were optimized. The kinetic behaviors of FAM-apt and GO, as well as the FAM-apt in homogeneous GO solution with CCRF-CEM cells, were investigated by monitoring the fluorescence intensity as a function of quenching and recovery time (Fig. 5a, b). As shown in Fig. 5a, the fluorescence quenching of FAM-apt as a function of incubation time in the presence of GO can be observed. The FAM-apt rapidly adsorbs to the surface of the GO and, after that, undergoes energy transfer, and at the same time, the fluorescence intensity is significantly reduced and tends to slow after 2 min. In contrast, CEM-apt is formed and the release from the GO surface is slower. The fluorescence intensity reached a platform when the incubation time was higher than 30 min (Fig. 5b). These time-dependent experiments show that GO, as an excellent quencher, rapidly quenches FAM-apt fluorescence and gradually regains fluorescence in the presence of CEM.

In order to make the fluorescent aptasensor more sensitive to the detection of CCRF-CEM, the reaction system used to optimize the GO concentration becomes indispensable. Figure 5c, which clearly illustrates our strategy, shows the effect of different concentrations of GO on the fluorescence intensity of FAM-apt in the absence (Fig. 5c, curve a) and in the presence (Fig. 5c, curve b) of CCRF-CEM. As we have seen from Fig. 5c, upon the addition of GO, the fluorescence signal background is significantly reduced. Figure 5d shows the restored fluorescence of the FAM-apt by 1 × 10^6^ CEM cells as a function of GO concentration. From Fig. 5d, we can find that when the GO concentration is 20 μg/mL, the ratio of *F*/*F*_0_ (where *F*_0_ and *F* are the fluorescence intensities of FAM at 518 nm in the absence and presence of CCRF-CEM, respectively) gets the highest value, which is 13.0354. Therefore, 20 μg/mL was considered to be the optimal GO concentration.

### CCRF-CEM Detection with GO-apt Fluorescent Aptasensor

**Fig. 6:**
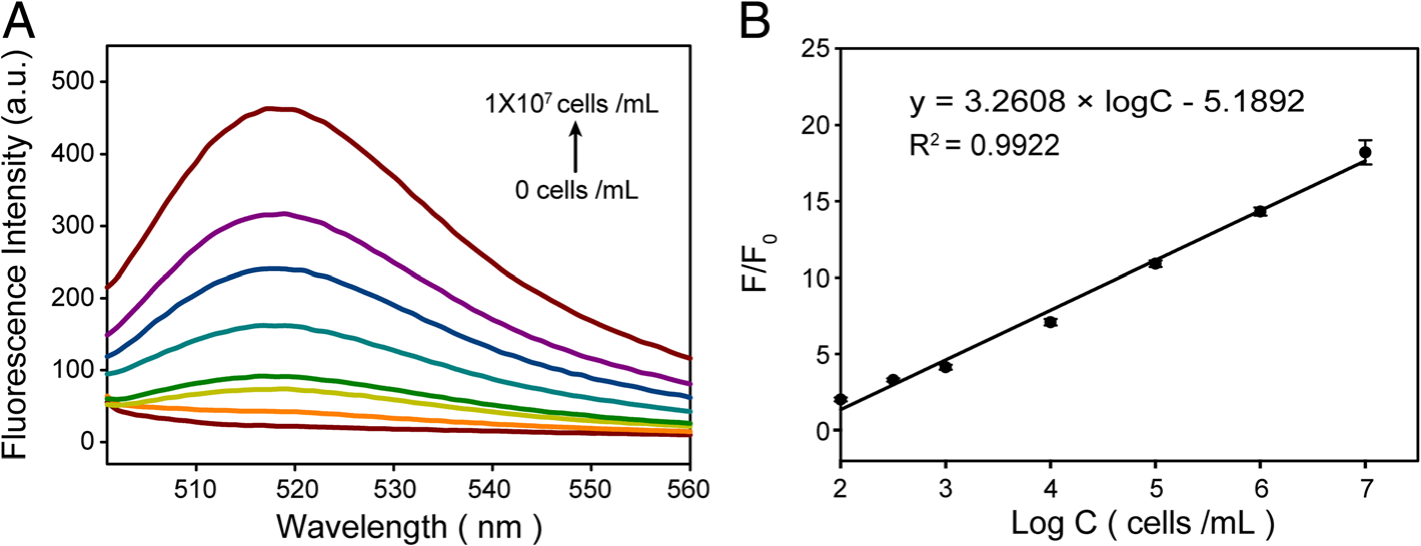
Go-apt detection of CEM cells. **a** Fluorescence emission spectra of GO-apt fluorescent aptasensor in the presence of different concentrations of CCRF-CEM cells. **b** Linear relationship between the fluorescence intensity rate (*F*/*F*_0_) and the concentration of CCRF-CEM cells

In order to obtain good experimental results, optimal experimental conditions were used to detect CCRF-CEM. Figure 6a shows that with the increasing number of CCRF-CEM from 0 to 1 × 10^7^, the fluorescence intensity is also increased accordingly. Furthermore, the *F*/*F*_0_ shows a clear linear dependence on the number of CCRF-CEM in the range of 1 × 10^2^–1 × 10^7^ (Fig. 6b). The linear regression equation is *Y*(*F*/*F*_0_) = 3.2608 × log *C* − 5.1892 (where *C* is the number of CCRF-CEM) with the regression coefficient *R*^2^ = 0.9922. The limit of detection is regarded as less than ten cells. Therefore, GO-based fluorescence aptamer sensing has a wide detection range so that can be used as an ideal biosensor to detect CCRF-CEM. Compared with the other methods, this method has higher sensitivity (Table 1) [34–39].

**Table 1:** Comparison of analytical properties for CCRF-CEM cytosensors

Detection method	Linear range	Detection limit (/mL)	Reference
Colorimetric	3.30 × 10^3^–2.69 × 10^3^	214	[34]
Fluorescence	1.00 × 10^3^–1.00 × 10^5^	250	[35]
Quartz crystal microbalance	8.00 × 10^3^–1.00 × 10^5^	8000	[36]
Electrochemical impedance spectroscopy	1.00 × 10^3^–1.00 × 10^7^	1000	[37]
Flow cytometry	7.50 × 10^3^–6.25 × 10^5^	750	[38]
Fluorescence	4.00 × 10^2^–5 × 10^6^	400	[39]
Fluorescence	1.00 × 10^2^–1 × 10^7^	10	This work

### Specificity of GO-apt Fluorescent Aptasensor

**Fig. 7:**
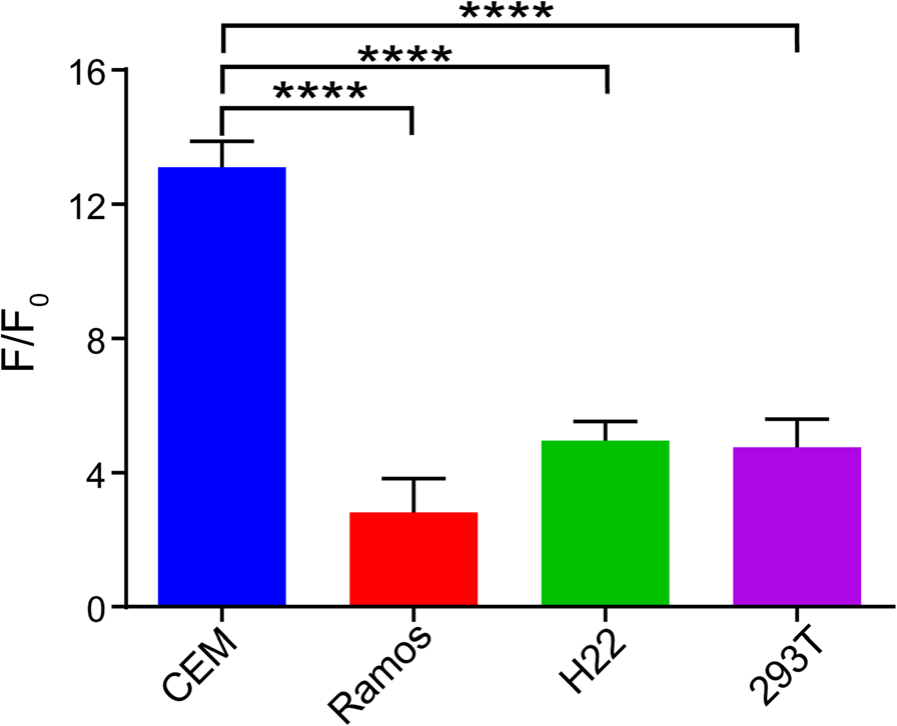
Specificity of the fluorescent aptasensor for CEM. The fluorescence intensity rate (*F*/*F*_0_) of GO-apt fluorescent aptasensor in the presence of CEM cells, Ramos cells, H22 cells, and 293T cells, respectively (1 × 10^6^), where *F*_0_ and *F* are the fluorescence intensity without and with detection cells at 518 nm. Excitation 490 nm

To investigate the specificity of GO-apt fluorescent adapters, several different cells were used to test the system, such as Ramos cells, H22 cells, and 293T cells. Each of the 100-μL reaction systems included 1 × 10^6^ cells. Figure 7 shows that CCRF-CEM gets higher fluorescence intensity than the other control groups. The results also clearly indicated that the designed fluorescent aptasensor embraced to be highly specific.

## Conclusions

We have developed a convenient, low-cost, and highly sensitive fluorescent aptasensor for detection of CCRF-CEM cells. This strategy cleverly uses the non-covalent bond interaction by the *π*-*π* stacking between graphene and single-stranded DNA and the superior performance of graphene-quenching fluorescence. Compared with the aptamer, the binding of the CEM-aptamer complex to GO is weak, so the fluorescence quenched by the graphene can be gradually restored. Under optimized conditions, the limit of detection is regarded as less than 100 cells. Therefore, based on its excellent performance, the fluorescent aptasensor has a broad prospect in tumor cell detection.

## Abbreviations

AFM: Atomic force microscopy
CEM-apt: CCRF-CEM-aptamer complex
FAM-apt: FAM-labeled Sgc8 aptamer
FRET: Fluorescence resonance energy transfer
GO: Graphene oxide
GO-apt: Graphene oxide-aptamer complex
PBS: Phosphate-buffered saline
