# Correction to: A Graphene Oxide-Based Fluorescent Aptasensor for the Turn-on Detection of CCRF-CEM

**DOI:** 10.1186/s11671-018-2525-2

**Published:** 2018-04-24

**Authors:** Jie Tan, Zongqiang Lai, Liping Zhong, Zhenghua Zhang, Rong Zheng, Jing Su, Yong Huang, Panpan Huang, Hui Song, Nuo Yang, Sufang Zhou, Yongxiang Zhao

**Affiliations:** National Center for International Research of Biological Targeting Diagnosis and Therapy, Guangxi Key Laboratory of Biological Targeting Diagnosis and Therapy Research, Collaborative Innovation Center for Targeting Tumor Diagnosis and Therapy, Guangxi Medical University, Nanning, 530021 Guangxi China

## Correction

In the original publication of this article [1] the author Liping Zhong was omitted. In this correction article the author and the corresponding details are provided. The publisher apologizes to the readers and authors for the inconvenience.

The original publication has been corrected.

## References

[1] Tan J, Lai Z, Zhong L (2018). Nanoscale Res Lett.

